# A novel method for purifying bluetongue virus with high purity by co-immunoprecipitation with agarose protein A

**DOI:** 10.1186/1743-422X-7-126

**Published:** 2010-06-13

**Authors:** Song Zhen, Dong Changyuan, Wang Lulu, Chen Dong-E, Bi Guoming, Dai Ming, Liu Jun

**Affiliations:** 1 Lab. of Molecular Virus & Cancer, State Key Laboratory of Virology, Wuhan University School of Basic Medicine, Wuhan University, Wuhan 430071, China; 2 Dept. of Epidemiology & Health Statistics, Wuhan University School of Public Hygienics, Wuhan University, Wuhan 430071, China

## Abstract

**Background:**

Bluetongue virus (BTV) is an icosahedral non-enveloped virus within the genus *Orbivirus *of *Reoviridae *and exists as 24 distinct serotypes. BTV can infect all ruminant species and causes severe sickness in sheep. Recently, it was reported that BTV can infect some human cancer cells selectively. Because of the important oncolysis of this virus, we developed a novel purifying method for large-scale production. The purifying logic is simple, which is picking out all the components unwanted and the left is what we want. The process can be summarized in 4 steps: centrifugation, pulling down cell debrises and soluble proteins by co-immunoprecipitation with agarose Protein A, dialysis and filtration sterilization after concentration.

**Results:**

The result of transmission electron microscope (TEM) observation showed that the sample of purified virus has a very clear background and the virions still kept intact. The result of 50% tissue culture infective dose (TCID_50_) assay showed that the bioactivity of purified virus is relatively high.

**Conclusions:**

This method can purify BTV-10 with high quality and high biological activity on large-scale production. It also can be used for purifying other BTV serotypes.

## Background

Bluetongue virus (BTV) is an icosahedral non-enveloped virus within the genus *Orbivirus *of *Reoviridae *[1] and exists as 24 distinct serotypes [2]. BTV can infect ruminant species mainly through the biting *Culicoides *species [3]. Particularly, it usually causes severe bluetongue diseases (BT) only in sheep and some species of deer. Over the past century, BTV has never shown infectivity on *Homosapiens*, and no normal human being cells have been successfully infected by BTV [4].

The genome of BTV contains ten linear segments of double-stranded RNA, and each of them codes one of the viral proteins. The virion of BTV has two protein shells with about 850-Å-diameter. The innermost shell is composed of 120 copies of protein VP3 (about 103 KD), which encloses three proteins (VP1, VP4 and VP6) and 10 segments of dsRNA genome, while the outer shell consists of protein VP2 (111 KD) and protein VP5 (about 59 KD). The two shells are linked by 780 copies of protein VP7 (about 38 KD) [5].

Protein VP2, coded by L2 segment, is the major cell adhesion protein [6-8] and the most variable protein in BTV. It can induce neutralizing antibody in the infected host. On the basis of the antigenicity of protein VP2, all of the 24 serotypes of BTV can be distinguished [9,10]. Besides, Protein VP5 helps to control the serotype of BTV [11].

Recently, BTV-10 has been reported that it can infect some human cancer cells selectively. The viral dose-dependent cytopathic effect (CPE) can be effectively induced by both virion amplification and virus-induced apoptosis on human liver carcinoma cell line (Hep-3B) and human lung carcinoma cell line (A549), while no visible CPE could be observed or detected in primary human embryo lung fibroblast cell (HEL) even after 5th day post-infection [4]. Another study about an unserotyped BTV, which was isolated from Xiangfan, Hubei province of china in 1990 and named BTV-HbC_3_, found that it can cause apoptosis on Hep-3B cells and paraptosis on A549 cells [12]. Further more, in the in vivo test this strain can make MA782-induced subcutaneously grown breast adenocarcinoma significantly regressed in mice model [13].

There are many reports about the methods for BTV purification, such as CsCl or saccharose density-gradient centrifugation. But these methods could hardly produce large amount of BTV virions and make BTV somewhat degraded [14-16]. We developed an effective method for high-throughput purifying BTV with high bioactivity, which is very helpful for the study of BTV anti-tumor effect since such studies need plenty of BTV with high bioactivity.

## Results

### Purity and integrity of purified BTV-10 by transmission electron microscopy (TEM)

**Figure 1:**
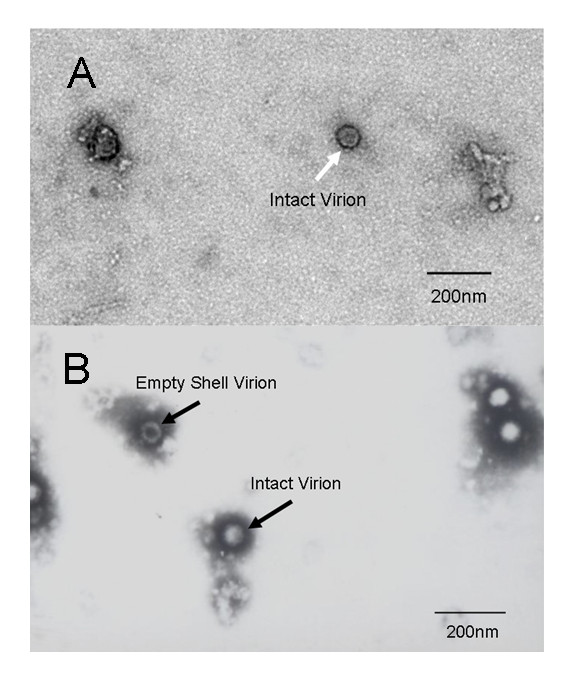
**Transmission electron microscopy pictures of the unpurified**. The sample was a direct collection of the virus culture on Vero cells. Picture A was photographed by digital camera and the virions were marketed by arrows. Picture B was photographed by film and the virions were marketed by arrows. In picture B, an empty shell was observed. Cell debris was also clearly observed in the two pictures.

**Figure 2:**
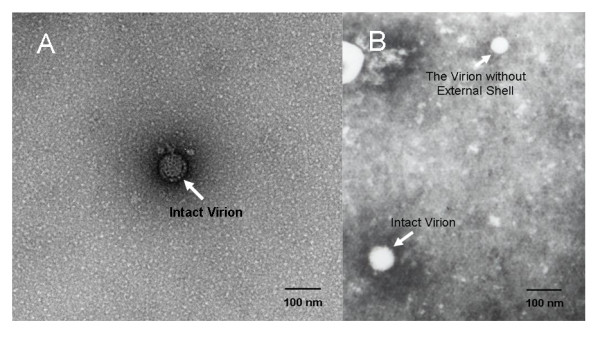
**Transmission electron microscopy pictures of the purified**. Picture A was photographed by digital camera and the virions were marketed by arrows. The two-layer structure of BTV virion was perfectly shown in picture A. Picture B was photographed by film and the virions were marketed by arrows. In picture B, an uncoated BTV virion was observed and was marked by arrow.

The photographs from TEM observation showed both unpurified (Fig. 1) and purified (Fig. 2) negatively stained samples. In the photograph of unpurified virus, limited amount of virions can be observed and they were surrounded with a mass of cell debris (Fig. 1A &1B). On the contrary, the photograph of purified sample revealed the purity and integrity of virus. In fact, the virions can be clearly observed in a clean background; meanwhile, the purified viral particles were still kept intact (Fig. 2A &2B).

### Biological activity test by TCID_50 _assay

**Figure 3:**
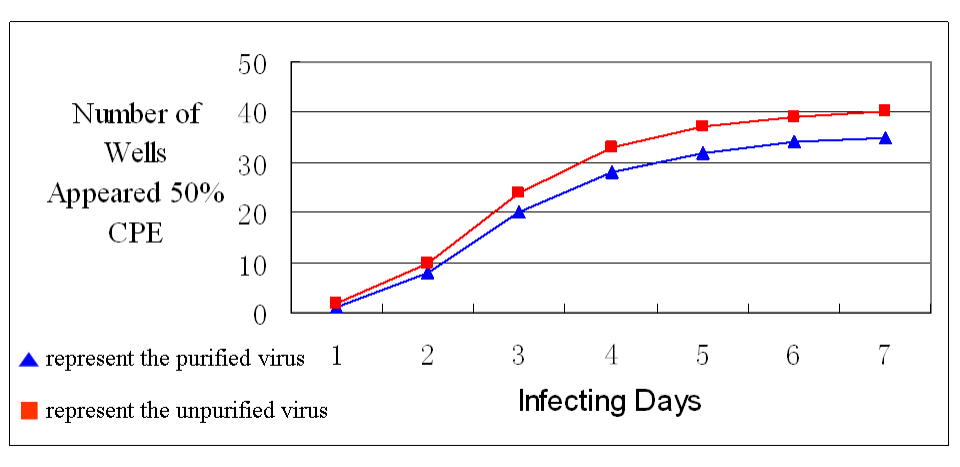
**The activity of purified and unpurified virus**. The chart graph shows the process of 50% cells exhibiting CPE as the infected time goes on according to the number of wells. The unpurified virus was used as a criterion to measure whether the purified virus was still active enough. As the infecting days went by, the number of wells appeared 50% CPE was increasing. The curvilinear trend of purified virus is in accordance with unpurified virus, and the wells exhibiting 50% CPE in purified virus per day is slightly less than that of unpurified virus.

The biological infectivity of purified and unpurified BTV was compared by the infectivity curvilinear trend and a TCID_50 _assay. Fig. 3 showed the process of 50% cells exhibiting CPE as the infected time goes on according to the number of wells, and as the curvilinear trend shown the infectivity of purified virus is similar to the unpurified virus. The TCID_50 _was calculated at the seventh day when the cells being infected. The TCID_50 _of unpurified BTV was 10^6.83 ^TCID_50_/ml and purified BTV was 10^6.42 ^TCID_50_/ml under the same conditions.

## Discussion

Nowadays, the research on anti-tumor mediated by virus is a hot field. Several viruses have been reported that they can infect tumor cell lines obviously, such as Reovirus [17] and Newcastle disease virus [18]. Over the past century, BTV has never been reported infectivity on *Homosapiens*, and no normal human being cells have been infected by this virus. We have studied BTV on anti-tumor for a long time and found that it can infect A549 and Hep-3B cell lines in vitro [4] and MA782 cell line in vivo. However, further study needs a large amount of virions with high biological activities, and current virus purification method can not meet our needs of researching.

This study introduced a novel method for purifying BTV-10 by cell debris co-immunoprecipitation with agarose Protein A. The logic is pulling out all the components unwanted, and then the left is what we want. The whole process can be summarized as three purification steps as well as one condensation and filtration sterilization step. The culture including Vero cells and the nutrient medium, which is used to proliferate BTV, were used as antigen to inoculate experimental rabbits in order to acquire polyclonal antibody. These antibodies containing various components were purified twice and then absorbed by protein A formerly bounded on agarose. At the same time, the culture mixture, containing lysed Vero cells, was centrifuged so as to remove some cell apparatus and big cell fragments. This step is very important and can be regarded as the first step of purification. Then the virus supernatant was mixed with the antibody-protein A-agarose complex. In this second step, molecules and cell pieces, which have antigenicity, have been pulled down mostly, whereas BTV virions still kept in the supernatant. But the second step is still not enough. In the supernatant, there were still a mass of molecules with no immunogenicity and those molecules that having antigenicity but did not induce immune response. So dialysis was needed, as the third step, to remove these ingredients. Thanks to the diameter of BTV particles is 850Å, it is safe enough to use dialysis tubing with 300 KD filtering, which can effectively remove all of the left molecules. After this third step, there were just BTV virions and small amount of microorganisms left. And the final step was the condensation of the virus solution by Polyethylene Glycol (PEG-20000) and sterilization filtration by 0.1 μ membrane filter.

The photographs about TEM observation on purified virus showed clearly that the purified viral particles were intact with a clear and clean background, while the unpurified sample's photographs showed few virions that were surrounded by a large amount of cell debris. This phenomenon means the virions were successfully separated from a mass of castoff.

Biological activity of purified viruses was tested by a TCID_50 _assay in Vero cells. Compared with unpurified virus, the TCID_50_/ml of the purified fell slightly. This falling may be caused by filtration sterilization. Considering that mycoplasma, which is a kind of potential pollutant in purification process, can penetrate 0.22 μ filter membrane, and the diameter of filter membrane we used for sterilization is 0.1 μ, which is very close to the diameter of a BTV particle, so as to avoid the purified virus contaminated by mycoplasma as much as possible. Unfortunately, this step may cause virions loss.

The purification process completely focuses on how to remove impurities with no regard to BTV serotypes, which is just like centrifugation method but can avoid physical injury on virions effectively. Thus it can be used for all BTV serotype's purification and we have purified several serotypes of BTV with this method (data not shown).

## Conclusions

After the above analysis based on the experiment, a new BTV purification method with high quality and high biological activity was established, and this may significantly push forward the research of BTV on anti-tumor and bluetongue sickness.

## Methods

### BTV-10 proliferation

BTV-10 and other serotype strains were acquired from China Animal Health and Epidemiology Center. Vero cell line was cultured in MEM (Gibco, USA) with 5% fetal bovine serum at 37°C, 5% CO2. When cells formed monolayer, culture medium was discarded and cells were washed by Phosphate Buffered Saline (PBS) (50 mM PB, 100 mM NaCl, 1 mM EDTA, pH7.4) twice. Subsequently, the monolayer of Vero cells were infected with 0.5 ml BTV-10 suspension (10^5 ^TCID_50_/ml) and incubated at 37°C for 2 hours. After that, 6-10 ml MEM with 10% fetal bovine serum was added, and cells were further incubated at 37°C. When 90% cells appeared CPE, the culture mixture was subjected to three rapid freeze/thaw cycles by putting it into liquid nitrogen and 37°C water bath. Then the virus-cell mixture (15 ml) was centrifuged at 5000 g for 10 minutes at 4°C and the supernatant was collected. We took out 5 ml supernatant (virion-cell debris) and preserved it at -20°C as control virus. The left virion-cell debris suspension was centrifuged at 12,000 × g for 10 minutes at 4°C. And then the supernatant was harvested and stored at -20°C. The uninfected cells were cultured in the same conditions as the control, which were used for producing polyclonal antibody either.

### Preparation of Antibodies

The Vero cell-culture mixture was subjected to three rapid freeze/thaw cycles by putting it into liquid nitrogen and 37°C water bath. Following ultrasonication for three 1-minute cycles in ice-bath, the cell debris suspension was centrifuged at 5000 × g for 10 minutes at 4°C. Subsequently, the supernatant was used as the antigen injected into experimental rabbits to produce polyclonal antibody.

The first inoculation was performed with 0.2 ml antigen and Freund's incomplete adjuvant (FIA) mixture and BCG vaccine (Bacillus Calmette-Guerin) to final concentration of 10 mg/ml. 2 weeks later, the second inoculation was performed the same dose. Then after 2 weeks, antigen without Freund's adjuvant was inoculated 3 days continuously (once a day) with 0.1 ml, 0.2 ml and 0.3 ml respectively as consolidating immunization. One week later, the blood was collected from experimental rabbits' heart and then the serum was harvested. The first purification of the antibodies was precipitation by saturated (NH_4_)_2_SO_4_. The sediment was resolved in 0.9% NaCl solution and was dialyzed by PBS at 4°C and the dialysis buffer was changed every 6 hours until the (NH_4_)_2_SO_4 _was completely removed. The trace amount of (NH_4_)_2_SO_4 _was checked by BaCl_2 _solution. Then the polyclonal antibody was further purified by Montage^® ^Antibody Purification K with PROSEP-A media.

### Purification of BTV-10 by Reverse Co-immunoprecipitation with Agarose Protein A

2000 μl antibodies (2 mg/ml) were mixed with 3 ml agarose Protein A completely and incubated at 4°C overnight. Then the mixture was centrifuged at 1000 × g for 10 minutes at 4°C. The supernatant was discarded while the deposit was collected and put into 10 ml unpurified BTV-10 supernatant. The deposit, containing antibodies and agarose Protein A, and BTV-10 suspension was incubated at 4°C overnight. After incubation, the mixture was centrifuged at 1000 × g for 10 minutes at 4°C.

### Purification of BTV-10 by dialysis

Following the preceding step, the supernatant was transferred into dialysis tubing with 300 KD molecular weight cutoff (MWCO) and was dialyzed by PBS at 4°C for 6 hours, which is one of the cell friendly buffers. Then, the dialyzed solution is concentrated by PEG-20000 and desalted by dialyzing at the same buffer again. This program was repeated 4 times.

### Transmission Electron microscopy assay

The purified BTV suspension was sterilized by filtration using 0.1 μ membrane filter firstly. Then both of the purified and unpurified virus suspension were negatively stained with 2% phosphotungstic acid. They were observed by electron microscope to compare the purity and integrity of viruses.

### Identification of biological activity of purified viruses

BTV TCID_50 _assay was used to test biological activity of BTV in Vero cells. Vero cells were cultured in two plates of 96 wells. When Vero cells formed monolayer, in one plate, they were infected with the purified virus suspension formerly sterilized by filtration; in the other plate, the cells were infected with the unpurified virus suspension. Both purified and unpurified virus suspensions were diluted 10 times for six serial dilutions arrange from 10^-3^-10^-8^. Each dilution was added to 12 wells with 100 μl/well. Meanwhile 24 wells (F line and G line) of cells of each plate were cultured as the control group. The two plates were placed at 37°C with 5% CO_2 _and CPE was observed everyday for 7 days. And TCID_50 _was calculated using Karber formula.

## Competing interests

The authors declare that they have no competing interests.

## Authors' contributions

SZ carried out Preparation of Antibodies, dialysis, concentration, drafted the manuscript and participated in TEM observation. DCY as the corresponding author designed the idea of the method and participated in TEM observation. CDE and WLL carried out the biological activity test, and WLL participated in revising the manuscript. Bi Guoming carried out the Reverse Co-immunoprecipitation; DM and LJ carried out BTV proliferation. All authors have read and approved the final manuscript.
